# Partitioning Analysis of Disparities and Time Trends in Alzheimer’s Disease and Related Dementia

**DOI:** 10.1093/geroni/igaa057.3121

**Published:** 2020-12-16

**Authors:** Igor Akushevich

**Affiliations:** Duke University, Durham, North Carolina, United States

## Abstract

This study uses Medicare data to i) identify age patterns, time trends, and race/geography-related disparities in prevalence and mortality of AD/ADRD; ii) apply partitioning methodology to separate out trends in causal components including incidence and survival, and iii) expand the method for analysis of disparities to identify the magnitude and trends in causal components of race-related disparities in AD/ADRD. Analysis shows that the trend in AD/ADRD incidence explains up to 70% of the observed changes in AD prevalence and mortality and is the main contributor to the differences in race-specific prevalence (higher for African Americans (AA)). Differences in race-specific incidence explain up to 80% of the disparity while 20% are due to difference in survival. This indicates that for AAs, incidence is worse but survival is better. This is confirmed by direct evaluations of hazard ratios: AD incidence HR for AA is 1.50(1.46,1.54) and 0.93(0.91,0.96) for survival after AD diagnosis.

